# Counterfactual Choice and Learning in a Neural Network Centered on Human Lateral Frontopolar Cortex

**DOI:** 10.1371/journal.pbio.1001093

**Published:** 2011-06-28

**Authors:** Erie D. Boorman, Timothy E. Behrens, Matthew F. Rushworth

**Affiliations:** 1 Department of Experimental Psychology, University of Oxford, Oxford, United Kingdom; 2 Centre for Functional Magnetic Resonance Imaging of the Brain, University of Oxford, John Radcliffe Hospital, Oxford, United Kingdom; Duke University Medical Center, United States of America

## Abstract

Decision making and learning in a real-world context require organisms to track not only the choices they make and the outcomes that follow but also other untaken, or counterfactual, choices and their outcomes. Although the neural system responsible for tracking the value of choices actually taken is increasingly well understood, whether a neural system tracks counterfactual information is currently unclear. Using a three-alternative decision-making task, a Bayesian reinforcement-learning algorithm, and fMRI, we investigated the coding of counterfactual choices and prediction errors in the human brain. Rather than representing evidence favoring multiple counterfactual choices, lateral frontal polar cortex (lFPC), dorsomedial frontal cortex (DMFC), and posteromedial cortex (PMC) encode the reward-based evidence favoring the *best* counterfactual option at future decisions. In addition to encoding counterfactual reward expectations, the network carries a signal for learning about counterfactual options when feedback is available—a counterfactual prediction error. Unlike other brain regions that have been associated with the processing of counterfactual outcomes, counterfactual prediction errors within the identified network cannot be related to regret theory. Furthermore, individual variation in counterfactual choice-related activity and prediction error-related activity, respectively, predicts variation in the propensity to switch to profitable choices in the future and the ability to learn from hypothetical feedback. Taken together, these data provide both neural and behavioral evidence to support the existence of a previously unidentified neural system responsible for tracking both counterfactual choice options and their outcomes.

## Author Summary

Reinforcement learning (RL) models, which formally describe how we learn from direct experience, can explain a diverse array of animal behavior. Considering alternative outcomes that could have been obtained but were not falls outside the purview of traditional RL models. However, such counterfactual thinking can considerably accelerate learning in real-world contexts, ranging from foraging in the wild to investing in financial markets. In this study, we show that three brain regions in humans (frontopolar, dorsomedial frontal, and posteromedial cortex) play a special role in tracking “what might have been”, and whether it is worth choosing such foregone options in the future. These regions encode the net benefit of choosing the next-best alternative in the future, suggesting that the next-best alternative may be privileged over inferior alternatives in the human brain. When people subsequently witness feedback indicating what would have happened had they made a different choice, these same regions encode a key learning signal—a prediction error that signals the discrepancy between what would have happened and what people believed could have happened. Further analysis indicates these brain regions exploit counterfactual information to guide future changes in behavior. Such functions may be compromised in addiction and psychiatric conditions characterized by an inability to alter maladaptive behavior.

## Introduction

It is widely agreed that a network of brain areas centered on ventromedial prefrontal cortex (VMPFC) and including anterior and posterior cingulate cortex encodes the values of choices that are taken [1]–[3]. Such a representation is assumed to be important for two functions. First, a representation of choice value is needed for decision making [1]. Second, a representation of a choice's value is needed for comparison with the subsequently experienced outcome [4],[5]. The discrepancy between the two, called the prediction error, is thought to be fundamental for learning because it partly determines the degree to which future reward expectations for the choice should be revised [6],[7].

While it is essential for organisms to represent the value of the choices that they take, there may also be considerable adaptive advantages associated with representing the reward potential of choices that are untaken. We refer to such potential, but untaken, choices as counterfactual choices. Such representations may confer both decision-making and learning advantages. First, if the reward potential of such choices is maintained neurally, then the organism may be better able to choose them in the future when it is beneficial, even in the absence of learning. Second, such a representation would make it possible to learn valuable information about what would have ensued had another choice been taken without having to incur both the energetic and opportunity costs that making the choice would have entailed. These representations would therefore enable us to exploit valuable, otherwise discarded, information and in turn make superior decisions in complex environments ranging from foraging in the wild to investing in financial markets. Unlike regret-related influences on behavior, which can lead to suboptimal biases in decision making, learning from counterfactual prediction errors should lead to more optimal decision making.

**Figure 1:**
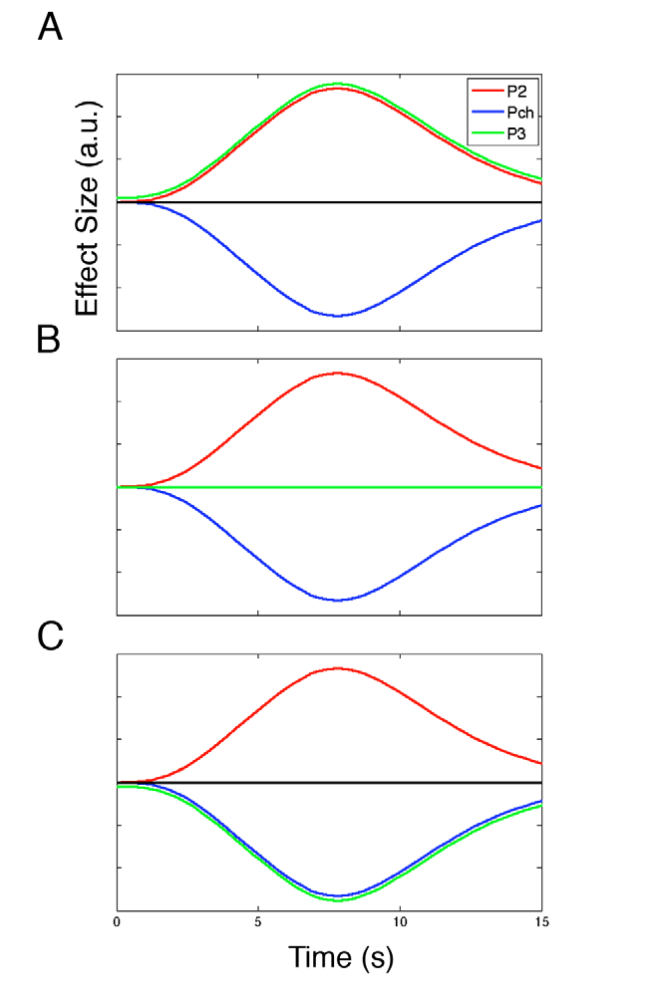
Theoretical LFPC coding schemes. Three hypothetical coding schemes for the LFPC are presented based on the findings of Boorman et al. (2009) [8]. (A) According to one possible scheme, there is a positive correlation with reward probabilities of both of the unchosen options and a negative correlation with the reward probability of the chosen option. This scheme might be expected if the LFPC encodes the average of the two unchosen options relative to the chosen option. (B) In the second hypothetical system, there is a positive correlation with the reward probability of the best unchosen option and a negative correlation with the reward probability of the chosen option, while the worst option is discarded altogether. This would be consistent with a system encoding the opportunity cost of the decision. (C) In the third scheme presented, the reward probability of the best unchosen option is encoded positively, while the reward probability of both of the alternatives—the chosen and worst unchosen options—are encoded negatively. This system would be useful for appraising the worthiness of switching to the best pending option. Pch, chosen reward probability; P2, highest unchosen reward probability; P3, lowest unchosen reward probability.

There is preliminary evidence that the lateral frontal polar cortex (lFPC) may contribute to such a decision-making representation during binary choice; lFPC activity increases with the potential future reward associated with the unchosen option [8]. Organisms, however, are frequently confronted with choices between multiple uncertain prospects, and whether and how lFPC activity might guide decision making in such situations is unknown. When a decision-making problem is no longer binary, several potential schemes for coding unchosen options emerge. First, lFPC may represent the potential future reward of both unchosen options (Figure 1A). There may, however, be limits to the number of potential alternative courses of action that can be represented [9],[10]. A second possible scheme, therefore, is that lFPC codes for the opportunity cost of the chosen option—that is, the value of the *best* of the unchosen options—and discards the worst option (Figure 1B). However, a third coding scheme is also possible; the FPC may weigh up the best unchosen option *relative* to the other options—in other words, relative to both the chosen option and the other unchosen option (Figure 1C). Such a system would be indicative of a mechanism for evaluating the merit of choosing the *best* pending option at forthcoming decisions rather than a system for evaluating whether it is beneficial to choose either pending option in the future. Such a coding scheme might allow for very efficient transitions in behavior in a changing environment, but as we explain below, it predicts more effective switching to some options than others.

There has been considerable recent interest in the possibility that the brain encodes fictive information [11],[12]. Specifically, it has been shown that activity in the dorsal striatum and parietal cortex is sensitive to the difference between the best possible outcome that could have been attained and the experiential outcome over gains but not losses [9]. Furthermore, single neurons in monkey dorsal anterior cingulate cortex are sensitive to the size of untaken outcomes [12]. It remains unclear, however, whether the brain encodes *prediction errors* for counterfactual choices—the discrepancy between the outcome for an untaken choice and the reward expectation associated with making that choice—in a separate and parallel manner to experiential prediction errors. This is necessarily difficult to establish in any paradigm in which there is a systematic relationship between the outcomes of counterfactual and experiential choices [11],[12].

To tackle these and related issues, we conducted an FMRI experiment in which human subjects made voluntary decisions between three options with independent reward probabilities, followed on most trials by decisions between the remaining two options that were unchosen during the first decision. Choices were made on the basis of two pieces of information: the probability of reward associated with each stimulus (which the participant had to estimate from recent outcomes of both chosen and unchosen options) and the reward magnitude associated with each stimulus (which was displayed on the screen beneath each stimulus and changed unpredictably from trial-to-trial). A Bayesian model was used to infer the reward outcome probabilities [13]. These manipulations enabled us to dissociate the relevant variables guiding immediate decisions (the three option *expected values*) from those guiding future decisions (the three option reward *probabilities*) and to test for the independent representation of counterfactual prediction errors during learning. Here we show that the lFPC, DMFC, and PMC encode key parameters for both selecting and learning about counterfactual options.

## Results

### Experimental Design

**Figure 2:**
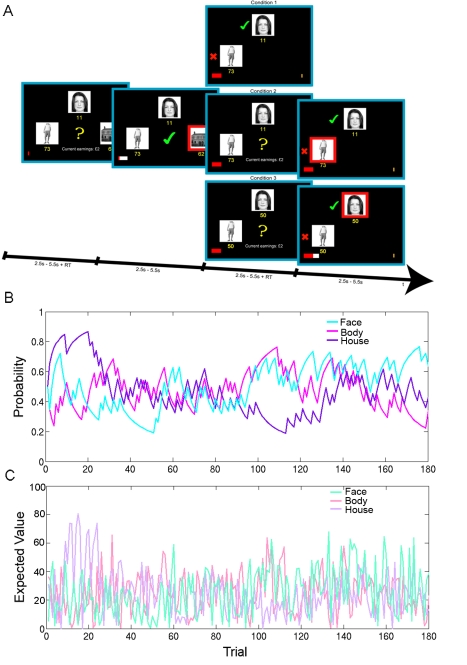
Experimental task, reward probabilities, and expected values. (A) Participants were faced with decisions between a face, whole body, and house stimulus, whose locations on the screen were randomized across trials. Participants were required to combine two pieces of information: the reward magnitude associated with each choice (which was shown in yellow beneath each stimulus) and the reward probability associated with each stimulus (which was not shown but could be estimated from the recent outcome history). When the yellow question mark appeared in the center of the screen, participants could indicate their choices by making right-hand button responses that corresponded to the location of the stimulus. The selected option was then highlighted by a red frame and the outcome was presented in the center of the screen: a green tick or a red X indicating a rewarded or unrewarded choice, respectively. If the choice was rewarded, the red points bar at the bottom of the screen updated towards the gold rectangular target in proportion to the number of points won. Each time the bar reached the target, participants were rewarded with £2. One of three conditions followed in pseudorandom order. In condition 1 the outcomes (rewarded or unrewarded) of the two unselected options were presented to the left of each stimulus, followed by the next trial. The points bar did not move in this condition. In conditions 2 and 3 participants had the opportunity to choose between the two options they had foregone at the first decision. In condition 2 the points associated with each stimulus remained the same as at the first decision. In condition 3 the points both changed to 50. In both conditions 2 and 3, when the yellow question mark appeared for the second time, participants indicated their second choices. This was followed immediately by feedback for both the chosen option, which once again was highlighted by a red frame, and the unchosen option, to the left of each stimulus. If the chosen option at the second decision was rewarded, the red points bar also moved towards the target in proportion to the number of points won. This second feedback phase was followed by presentation of the next trial. (B) The probability of reward associated with the face, body, and house stimuli as estimated by an optimal Bayesian learner (Experimental Procedures) are plotted over trials in cyan, magenta, and purple respectively. The underlying reward probabilities varied from trial-to-trial according to a fixed volatility. The reward probabilities associated with each option were de-correlated (Results; Figure 3A). (C) The expected value (reward probability×reward magnitude) associated with face, body, and house stimuli are plotted across trials in turquoise, light pink, and light purple, respectively. Reward magnitudes were selected so that the correlation between expected values was also limited.

Participants performed a decision-making task in which they repeatedly chose between a face, house, and body stimulus that were presented at one of three locations at random (Figure 2A). On each trial random integers between 1 and 100 were displayed beneath the stimuli that indicated the size of potential reward associated with selecting that option. Participants were informed that since these reward *magnitudes* were generated randomly on each trial, it was not advantageous to track them across trials. However, participants were not directly cued about the *probability* with which each option would be rewarded if chosen. Instead, participants were told that these reward probabilities depended only on the recent outcome history. To produce a changeable environment, these reward probabilities varied from trial to trial according to a fixed volatility [13] during the course of the experiment. On two-thirds of trials (conditions 2 and 3), participants encountered a second decision between the two options that were foregone at the first decision, a manipulation that enabled more accurate estimates of participants' own ranking of the two unchosen options. Following the second decision, feedback on chosen and counterfactual options was presented, thereby allowing us to search for neural correlates of counterfactual prediction errors. On the other third of trials (condition 1), there was no second decision; instead feedback on the two unselected options was presented (Figure 2A).

**Figure 3:**
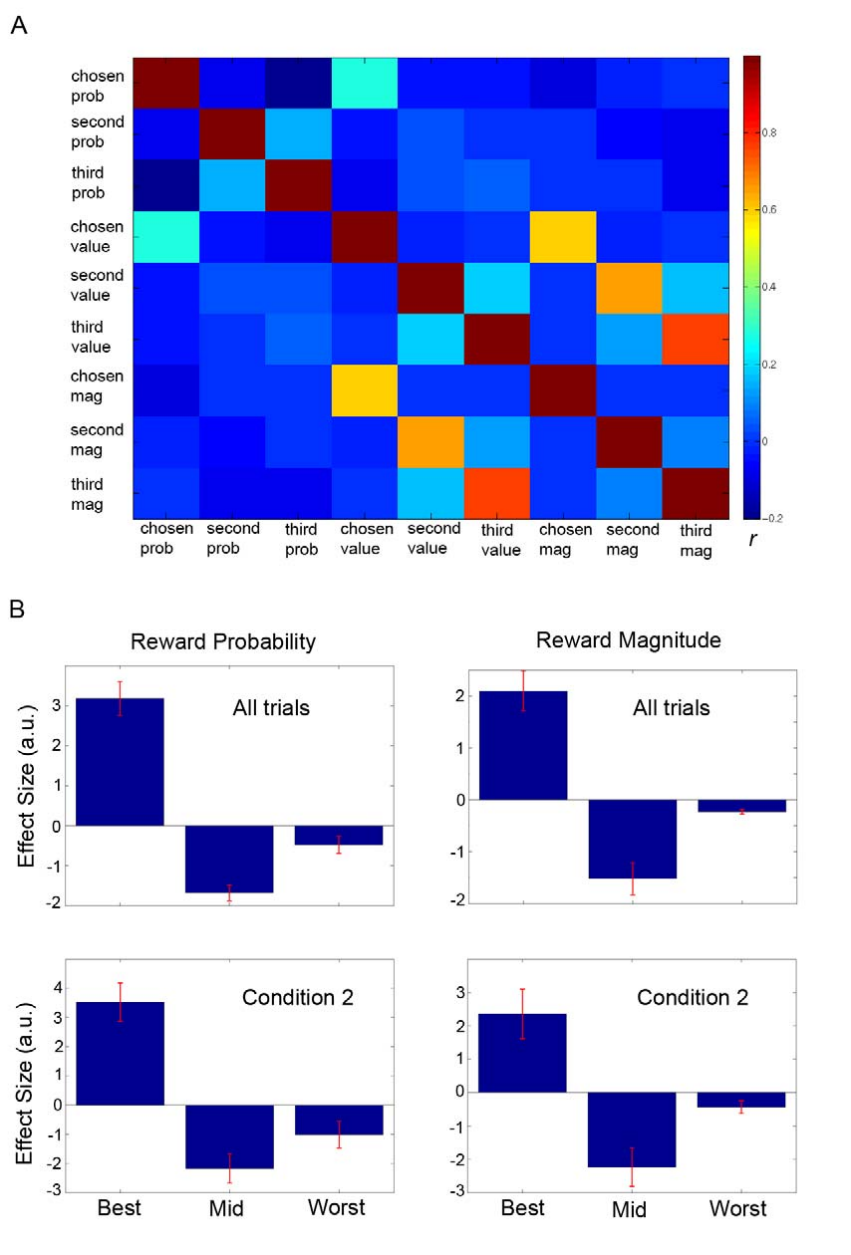
Cross-correlation matrix and behavioral regression coefficients. (A) Group cross-correlation matrix depicting mean correlation (*r*) across participants between reward probabilities, expected values, and reward magnitudes of chosen, next best, and worst options. (B) Mean regression coefficients (i.e., parameter estimates) related to the reward probabilities (left column) and reward magnitudes (right column) of the options with the highest, middle, and lowest expected value derived from a logistic regression on optimal choices (i.e., choices of the option with the highest expected value). Error bars represent standard error of the mean (s.e.m.).

An optimal Bayesian learner [13] was used to model participant estimates of the probabilities of reward associated with the options given the history of recent choice outcomes (i.e., rewarded or unrewarded chosen and unchosen options) (Figure 2B). The Bayesian learner enabled us to select a reward schedule that de-correlated the reward probabilities associated with each option. In the selected reward schedule, there was limited correlation between the reward probability associated with the three options (mean *r* across participants between body part and face stimuli = −.4; body part and house stimuli = 0.01; face and house stimuli = −.4) and between the expected value (reward probability×reward magnitude) associated with the three options (mean *r* between body part and face stimuli = −.2; body part and house stimuli = 0.03; face and house stimuli = −0.14). Although we could not know what choices our participants would ultimately make, this increased the likelihood that chosen and unchosen reward probabilities or expected values would also be de-correlated. As anticipated, there was indeed limited correlation between chosen and unchosen option reward probabilities (mean *r* for chosen and best unchosen <−.1; chosen and worst unchosen = −.2; best unchosen and worst unchosen = .37). Similarly, there was little correlation between the chosen and unchosen option values (mean *r* for chosen and best unchosen <.1; chosen and worst unchosen <.1; best unchosen and worst unchosen = .43) (Figure 3A). It is important to note that the random trial-by-trial fluctuations in reward magnitude meant that only option probabilities had to be maintained for making *future* choices. This feature of the experimental design also meant that it was optimal to learn about option reward probabilities but not reward magnitudes.

### Choice Behavior

Before searching for evidence of a neural representation of unchosen options, it is important to assess whether there is behavioral evidence that people not only update their future reward expectations experientially, from the feedback provided for the chosen option, but also counterfactually, from the feedback provided for unchosen options. Similarly, it is important to establish whether or not there is evidence that behavior is influenced by the values of the different possible options that might be taken.

To assess the fit to behavior of the optimal Bayesian model, which used both experiential and counterfactual feedback to update estimates of options' reward probabilities, we computed the log likelihood and Bayesian Information Criterion and compared the fit to two alternative models (Table 1; Figure S4). To test whether people *learn* from counterfactual feedback, we compared the fit of the optimal Bayesian model to the fit of an alternative model that we term an “experiential Bayesian model” because it is identical to the optimal model except that it does not update unchosen options. As can be seen from Table 1, the optimal Bayesian model clearly outperformed the experiential Bayesian model, implying that people learned from both experienced and counterfactual feedback. Finally, we also compared the optimal model with a conventional Rescorla Wagner model that updates both chosen and unchosen options. The optimal model was also a far better fit to behavior than the Rescorla Wagner model that updates both chosen and unchosen options (Table 1; Text S1). It is notable that these Bayesian models have some parallels with learning models used previously to analyze behavior during experimental games [14].

**Table 1:** Comparison of model fits for the optimal Bayesian, experiential Bayesian, and Rescorlar Wagner models.

Model	Parameters	Log Likelihood	BIC
Optimal Bayesian	3 (0 predictor)	−2,080	4,175.6
Experiential Bayesian	3 (0 predictor)	−2,265.8	4,547.1
Rescorla Wagner	4 (1 predictor)	−2,634.2	5,289.1

To further assess whether the optimal Bayesian model captured choice behavior, as well as which variables influenced participant choices, we performed logistic regression analyses. This analysis aimed to determine the degree to which choosing the most valuable option was influenced by the outcome probabilities as estimated by the optimal Bayesian model and reward magnitudes associated with the best, mid, and worst options (see Text S1 for details). This analysis revealed a strong positive effect of the best option (reward probability: *t*(18) = 7.56, *p*<0.0001; reward magnitude: *t*(18) = 5.45, *p*<0.0001), a strong negative effect of the mid option (reward probability: *t*(18) = −8.50, *p*<0.0001; reward magnitude: *t*(18) = −4.86, *p*<0.0001), and a modest but highly consistent effect of the worst option (reward probability: *t*(18) = −2.27, *p* = 0.02; reward magnitude: *t*(18) = −5.16, *p*<0.0001) on choices of the best option (i.e., optimal choices) (Figure 3B). In other words, the reward probabilities estimated by the optimal Bayesian model and the explicitly presented reward magnitudes were both strong predictors of participants' choices. Consistent with the experimental design, the reward magnitude from the previous trial by contrast did not have any impact on current choices of the best option (best reward magnitude trial *i-1*: *t*(18) = 0.49, *p* = 0.31; mid reward magnitude trial *i-1*: *t*(18) = 0.67, *p* = 0.25; worst reward magnitude trial *i-1*: *t*(18) = 0.59, *p* = 0.28). Thus, optimal estimates of reward probability and *current* but not past reward magnitudes strongly influenced participant behavior (see Text S1 for more details). The analyses described above indicate that although the best and mid options principally drive choice, the worst option also consistently explains a small amount of variance in participant choices of the best option.

Whether people learn differently from experiential and counterfactual feedback remains an open question. To address this we constructed an additional model in which separate learning rates scaled chosen and unchosen prediction errors (Text S1). These two participant-specific learning rates were fitted to each participant's choices using standard estimation procedures. In our experimental task, there was no difference between the learning rates for chosen and unchosen feedback (*t*(18)<.25, *p*>0.4), suggesting that people may not learn differently from counterfactual feedback and experiential feedback when these sources of information are both available and equally informative.

### The Reward-Based Evidence Favoring Future Switches to the Best Pending Option

**Figure 4:**
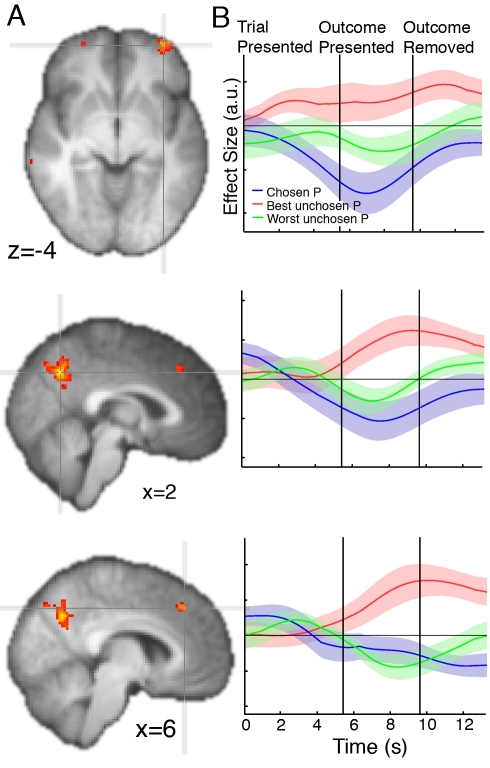
The reward-based evidence favoring future choices of the best pending option. (A) Axial and sagittal slices through z-statistic maps relating to the effect of reward probability of the unchosen option with the highest reward probability. Maps are thresholded at z>2.8, *p*<0.003 for display purposes, and are displayed according to radiological convention. (B) Time course of the effects of the reward probability for the chosen option (blue), the unchosen option with the highest reward probability (red), and the unchosen option with the lowest reward probability (green) are shown across the first decision-making and feedback phases. Time courses are not corrected for the hemodynamic lag. Thick lines: mean effect size. Shadows: ± s.e.m. Top row: LFPC; middle row: PMC; bottom row: DMFC.

In order to search for evidence of neural activity encoding the reward association of the best unchosen option, we first tested for voxels across the whole brain where activity correlated with the reward probability of the best unchosen option—one of the relevant metrics to track across trials to inform future switches. We also included the reward probability of the chosen and worse unchosen options as separate terms in the general linear model (Table S1). This analysis revealed three regions with a positive effect of the reward probability of the best unchosen option (Z>3.1, *p*<0.001 uncorrected; cluster extent >10 voxels): left lateral frontopolar cortex (lFPC; Z = 3.50, MNI x = −36, y = 58, z = −4; Z = 3.64, x = −32, y = 46, z = −2), posteromedial cortex (PMC; Z = 3.70, x = 2, y = −62, z = 38), and dorsomedial frontal cortex (DMFC; Z = 3.33, x = 6, y = 34, z = 42) (Figure 4A; Table S2). Although no activations exceeded the threshold in right lFPC, an activation emerged at the reduced threshold of *p*<0.003, uncorrected (see Figure 4A). It is important to note that we have deliberately refrained from using the most sensitive regressor in this analysis because the purpose was to define ROIs that will be unbiased for later tests. When the best unchosen probability *relative* to the chosen probability is instead used as the regressor, there is a more robust effect in the lFPC (Figure S1; Z = 3.99, MNI x = −36, 58, −6), as expected based on a previous demonstration that the lFPC encodes a relative signal in a binary choice task [8].

To test whether and how the lFPC, PMC, and DMFC might also encode alternative options, we performed orthogonal analyses on the time courses of these regions identified by the whole-brain analysis. In addition to a positive correlation with the best unchosen reward probability, the lFPC signal correlated negatively with the reward probability for both the chosen (*t*(18) = −3.46, *p*<0.005) and other unchosen option (*t*(18) = −2.21, *p*<0.03) during the decision-making phase (Figure 4B). The lFPC signal correlates positively with the reward potential of the best alternative and negatively with the reward potential of both the chosen option and the worse unchosen option, suggesting that lFPC forecasts the evidence in favor of choosing the better of the two unchosen options at future choices. Such an activity pattern is inconsistent with FPC simply maintaining a representation of the advantage to be gained from switching to any alternative action. It would, however, be predicted if lFPC represented only one alterative action in a pending state. Under such a scheme, the negative encoding of the reward probability of both the chosen option and the worse unchosen option can be interpreted as reflecting the potential opportunity cost of foregoing the chosen action or the other alternative action if there were to be a switch in behavior to the pending state.

We also identified evidence for a very similar pattern of activity in PMC and a closely related one in DMFC. In PMC there was a significant negative correlation with the chosen option (*t*(18) = −2.23, *p*<0.02) and the other unchosen option during the decision-making phase (*t*(18) = −2.0, *p*<0.03), whereas in DMFC, there was a significant negative correlation with the chosen option (*t*(18) = −1.9, *p*<0.04), but the effect of the worse unchosen option was not significant (*t*(18) = −1.24, *p*>0.11) during the decision-making phase.

**Figure 5:**
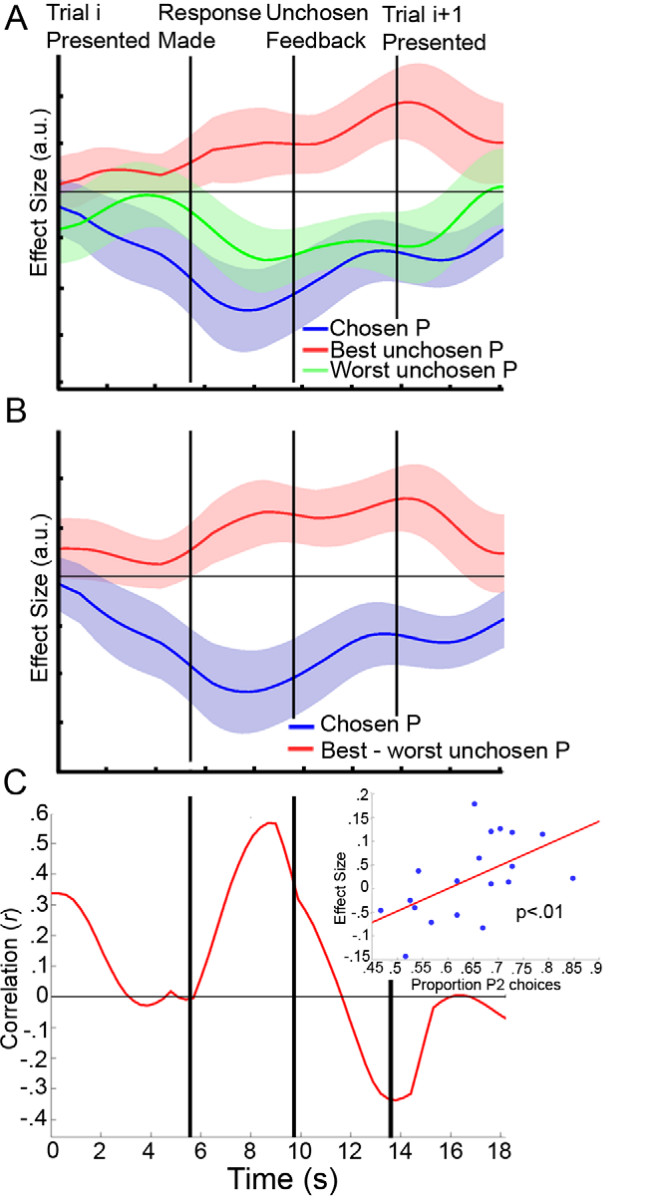
LFPC effect predicts individual differences in behavior. (A) Time course is plotted on the subset of trials during which there was no second decision across the entire trial. (B) Time course of LFPC effects of the best minus the worst unchosen probability (red) and the chosen probability (blue) in condition 1 are shown plotted across the trial. Conventions are the same as in Figure 4. (C) Between-subject correlation is plotted across the trial. The curve depicts the correlation (*r*) between the effect of the best minus the worst unchosen probability in the LFPC from conditions 2 and 3 (i.e., when there was a second decision) and the proportion of trials on which participants chose the option with the highest reward probability at the second decision. Inset: scatterplot of the effect size against the behavioral index at the time of the first peak in the effect of the best relative to the worst unchosen probability from condition 1 shown in (B). The time point selected for the scatterplot is thus unbiased with respect to the data used for the between-subject analysis.

We repeated these analyses on only those trials on which a single decision had to be made in order to exclude the possibility that activity related to a second decision could confound activity related to the feedback phase of a first decision. Because of the short temporal interval between the first feedback phase and the second decision-making phase in our experiment on some trials, activity related to late time points during the first feedback period is difficult to dissociate from activity related to the second decision in these time course analyses. When there is a second decision the effect of the worst unchosen option flips from being encoded negatively to positively when it frequently becomes the best (and only) unchosen option at the second decision-making period. To circumvent such issues, we reexamined the time course during condition 1 in which there was not a second decision that could interfere with the lFPC response to the first decision and feedback phases. Although the number of trials in this analysis is substantially reduced, there were still significant negative effects of the chosen probability (*t*(18) = −2.93, *p*<0.005) and worst unchosen probability (*t*(18) = −2.62, *p*<0.01) (Figure 5A) in lFPC. Similarly, the PMC signal was significantly and negatively correlated with the chosen reward probability (*t*(18) = −2.17, *p*<0.03) and worst unchosen reward probability (*t*(18) = −2.06, *p*<0.03). However, in DMFC there was still only a significant negative effect of the chosen option (chosen option: *t*(18) = −2.36, *p*<0.02; worst unchosen option: *t*(18) = −0.98, *p*>0.17).

The interpretation that this signal reflects the future evidence in favor of choosing the best unchosen option at subsequent decisions makes a testable prediction about behavior. Participants in whom this evidence is better represented should go on to choose the best pending option more frequently. It is important to note that during the initial decision, it is the unchosen option with the highest reward probability that is likely to be the best option at second decisions. In accord with the hypothesis, the greater the effect of the best unchosen probability relative to the worst unchosen probability in the lFPC across participants in conditions 2 and 3 (i.e., when there was a second decision), the more frequently participants chose the pending option that was associated with the highest reward probability at second decisions (Figure 5C).

The identified lFPC coding scheme further suggests that people may be better at adapting behavior to the next best alternative than to the worse alternative when confronted with decisions between multiple options. Such a scheme makes an intriguing prediction about behavior. It is possible that people switch choices to the next best alternative more effectively than they do to the worse alternative. This prediction is testable in our task because the previously worst option might become the best option when random reward magnitudes are introduced at the onset of a new trial. Consistent with this proposition, we found that participants adapted choices to the best pending option when it was optimal significantly more frequently than they did to the worse pending option when it was optimal, even when the analysis was restricted to trials matched for value difference (*t*(18) = 2.17, *p* = 0.02).

### Counterfactual Prediction Errors

A valuable source of information during learning comes not only from the experienced outcomes of actions that are taken but also from the consequences of alternative potential actions that might be taken in the future. It was hypothesized that brain regions that encode future reward expectations related to unchosen options might also be involved in updating those expectations. This prediction is based upon recent evidence demonstrating that prediction error-like signals can be identified in brain regions thought to be specialized for visual and social processing when participants must their expectations during visual and social learning, respectively [15]–[22]. We reasoned that the same principle might hold true for learning about unchosen options.

**Figure 6:**
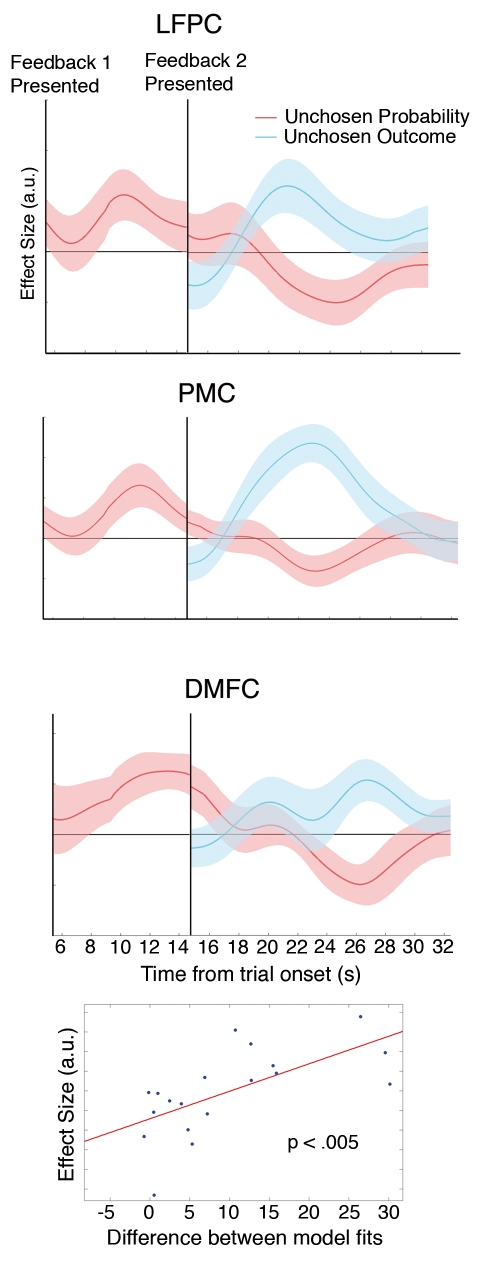
Counterfactual prediction errors. The time course of the fictive prediction error is plotted decomposed into its component parts: the expectation of reward for the unchosen option (pink) and the outcome of the unchosen option (cyan). The time course is plotted from the onset of the initial feedback for the first decision. There is a positive effect of the fictive outcome and a negative effect of the fictive expectation after the revelation of the outcome in each region. Conventions are the same as in Figure 4. Bottom row inset plots the counterfactual prediction error effect size in the PMC against the difference between the fit to behavior of the optimal and experiential Bayesian models, where each point represents a single subject.

Analysis of the time course of the lFPC, DMFC, and PMC regions identified by the whole-brain analysis revealed a significant correlation with the unchosen, but not chosen, prediction error following the delivery of feedback for the second decision in each region (*unchosen* prediction error: lFPC: *t*(18) = 2.01, *p*<0.05; DMFC: *t*(18) = 2.8, *p*<0.01; PMC: *t*(18) = 4.35, *p*<0.0005; *chosen* prediction error: lFPC: *t*(18) = .39, *p*>0.3; DMFC: *t*(18) = .29, *p*>0.35; PMC: *t*(18) = −0.11, *p*>0.45). Moreover, the pattern of activity in these regions elicited by counterfactual rewards (Figure 6) was similar to that displayed by dopaminergic neurons for experienced rewards [23]. Activity correlated positively with the probability of reward for the unchosen option before the outcome was revealed (lFPC: *t*(18) = 1.89, *p*<0.05; DMFC: *t*(18) = 3.55, *p*<0.005; PMC: *t*(18) = 2.06, *p*<0.05). Following the delivery of feedback, activity correlated negatively with this same probability (lFPC: *t*(18) = −1.78, *p*<0.05; DMFC: *t*(18) = −1.92, *p*<0.05; PMC: *t*(18) = −1.90, *p*<0.05) and positively with the unchosen outcome (lFPC: *t*(18) = 1.81, *p*<0.05; DMFC: *t*(18) = 2.65, *p*<0.01; PMC: *t*(18) = 4.31, *p*<0.0005). These regions' activity therefore reflected both components of the counterfactual prediction error—the counterfactual outcome minus the expectation (Figure 6). Replicating previous findings, we identified experiential reward prediction errors in the ventral striatum, among other regions (Figure S2; Table S2).

We further considered the possibility that neural counterfactual prediction error signals might have an impact on behavior. We reasoned that people in whom there is a greater effect of counterfactual prediction errors may learn more effectively from counterfactual feedback. To test this hypothesis, we compared the model fit to behavior of the optimal Bayesian model that updates both chosen and unchosen options and the experiential Bayesian model that only updates the chosen option. The difference in the fit to behavior of these models provided an index of the extent to which people learned from counterfactual feedback. Across the sample of participants, there was a tight relationship between the effect size for the counterfactual prediction error and the difference between the fits of the optimal and experiential models in the PMC (*r* = 0.64, *p*<0.005; Figure 6). There was a similar tendency in lFPC, though this did not reach statistical significance (lFPC: *r* = 0.39, *p* = 0.10), but not in DMFC (*r* = .24, *p*>0.3).

Regret should theoretically grow as the difference between the reward magnitude of the foregone outcome and the chosen outcome increases, independently of the reward expectation [24],[25]. Unlike regret-related FMRI signals that have been identified previously [11],[25], activity in the lFPC, DMFC, and PMC was not sensitive to the difference between the size of the outcomes for the unselected and the selected options (lFPC: *t*(18) = −0.44, *p*>0.3; DMFC: *t*(18) = −0.86, *p*>0.2; PMC: *t*(18) = −0.59, *p*>0.25). Taken together, these findings demonstrate that counterfactual prediction errors are dissociable from regret in the lFPC, DMFC, and PMC.

## Discussion

A number of brain regions have been implicated in the representation of value during learning and decision making [17],[26], but in general the focus has been on the choices that participants make and the rewards they experience. Within the frontal cortex, the orbitofrontal and anterior cingulate cortical regions have most often been the focus of such research. Here, however, we show that the lPFC maintains a representation of the net profit to be expected from choosing the next best alternative in the future. The lFPC BOLD signal increases as the probability of obtaining reward from the next best alternative increases. The reward that might be sacrificed by switching away from the current action may be represented as a cost of switching to the alternative; lFPC BOLD activity decreases as the probability of reward associated with the current course of action increases. Similarly, the reward that might be lost by foregoing the worst unchosen option can be conceived of as a cost; lFPC BOLD decreases as the probability of reward associated with the worst foregone option increases. When there are multiple alternatives to choose between, the pattern of lFPC activity is therefore consistent with a system that forecasts the reward potential of the best alternative option and the costs of not taking both the current course of action and the other alternative. This coding scheme is consistent with a system that accumulates evidence in favor of choosing the best pending option in the future so that it can be switched to effectively.

There were several plausible schemes according to which the lFPC could represent unchosen alternatives. The data presented here provide evidence in favor of the system depicted in Figure 1C—namely that the lFPC encodes the merit of potential future switches to the next best alternative. This interpretation is supported by a between-subject correlation between the effect of the reward probability associated with the best relative to the worst unchosen option in the lFPC and choices of the best pending option. It is also supported by the finding that participants are superior at adapting behavior to the next best alternative than to the worse alternative when these choices are optimal. The lFPC signal, as in a previous study [8], contained peaks during both the decision-making and feedback phases (see Figure 4A). This time course is consistent with the notion that the lFPC tracks the relevant decision variable across time for forthcoming choices.

Several accounts propose that the FPC maintains information across time for future deployment [9],[27]–[31]. FPC activity has been shown to increase when an intention or a task set has to be maintained during a delay and then implemented [28]–[30],[32],[33], while damage to left anterior prefrontal cortex, including left FPC, disrupts effective switching between task sets in such paradigms [34]. It was recently shown that monkey FPC encodes the decision (left or right response) over an extended delay around the time of feedback, particularly when it was advantageous to maintain this information for use on the next trial [35]. Furthermore, FPC is selectively recruited when participants must maintain information in working memory whilst performing a subtask for the purpose of using the pending information upon completion of the subtask [27], particularly when the time at which the pending information must be used is unpredictable [36]. On the basis of such findings, it has been posited that FPC has a special role in cognitive branching—the maintenance of pending information related to a previous behavioral episode during an ongoing behavioral episode for future use [9],[37]. Following this framework, in our experiment the current decision could be conceived of as the ongoing behavioral episode, and the best unchosen option as the pending information, which may be selected in the future. While our findings are consonant with such accounts, we have shown that the FPC not only represents pending information or intentions for future use, but that it encodes the *evidence* in favor of their future deployment. Moreover, we have demonstrated that when people are confronted with more than two alternatives, the FPC specifically encodes the evidence in favor of the unchosen alternative that would be most advantageous to be selected in the future, a finding consistent with the view that there may be limits to FPC coding during decision making [9].

In our experimental setup, participants should have expected to encounter a second decision on approximately two-thirds of trials. Despite this manipulation we found no evidence that this knowledge influenced participants' initial decisions (see Text S1, Experimental Procedures). It is nevertheless possible that participants anticipated having to make a second decision at which point reward magnitudes would either remain the same as they were or equate to 50. In a previous investigation in which participants made binary choices with no intervening second decision, the lFPC was shown to encode the unchosen option positively and the chosen option negatively, consistent with the positive coding of the next best alternative and negative coding of the chosen option we have revealed here in a multi-option context. It would be interesting to examine the coding of lFPC when people make decisions between multiple alternatives in the absence of any requirement to make decisions between the remaining unchosen options.

lFPC appears to be only one component of a network of areas that are interconnected and whose activity tracks the advantage to be gained from switching to the next best alternative. The activation in PMC may be in area 31 of the posterior cingulate cortex [38],[39], while the DMFC activation appears to be situated between the pre-SMA and dorsal anterior cingulate cortex (dACC). Anterograde and retrograde studies have examined the anatomical connections between these regions in monkeys. Area 31 of the monkey has reciprocal connections with both FPC and parts of DMFC [38], and FPC also projects to parts of DMFC [40]. A recent study in macaque monkeys has identified neurons in a neighboring region of the PMC that are selective for exploration and switching between four different response alternatives [41]. Moreover, the pre-SMA has been implicated in switching between task sets [42]. Taken together, these findings suggest that the lFPC, PMC, and DMFC regions might form part of an interconnected network dedicated to tracking the evidence in favor of future switches to the best pending option and, in collaboration with the mid-IPS, implementing such switches [8],[43]. It is notable that the three components of the counterfactual choice circuit are some distance from foci in ventral DMFC, ventral PMC, and VMPFC in which the BOLD signal is correlated with the value of the action that is chosen [1],[3],[8],[44]–[47].

Reinforcement-learning models theorize that agents should learn from both chosen and unchosen outcomes [6]. Nevertheless, to our knowledge *prediction error* signals related to unchosen options have yet to be identified in the mammalian brain. Lohrenz and colleagues [9] have reported activity in the dorsal striatum and posterior parietal cortex that they refer to as a fictive error signal. Although this metric influences behavior in interesting ways [11],[48], it is distinct from the one that we report here because it correlates with the best possible outcome that could have been attained minus the experienced outcome received, over gains but not losses. Crucially such fictive signals pertain to the choice of a different level of the taken action. They do not contain information about alternative actions with independent probabilities of success. By contrast, a counterfactual *prediction* error—the counterfactual outcome minus its *expectation*—should theoretically be proportional to the degree to which future reward expectations of unchosen options are updated. We found that the lFPC, DMFC, and PMC—regions whose activity is sensitive to the unchosen option with the highest reward probability during initial decisions—encoded counterfactual prediction errors when participants witnessed counterfactual outcomes of subsequent decisions.

A prediction error should theoretically signal the prediction of an event before its revelation and, following its revelation, the discrepancy between the event's occurrence (or non-occurrence) and the prediction—a prediction error [6]. It has been well documented, in the context of experienced rewards, that both signals are closely approximated by the firing rate of phasically active dopamine neurons [23]. The pattern of activity in lFPC, DMFC, and PMC similarly exhibited both of these components but in relation to counterfactual rewards: before the outcome was revealed there was a positive correlation with the expectation of reward for the unchosen option; once the outcome was witnessed, there was a negative correlation with this same expectation and a positive correlation with the outcome (reward or no reward for the unchosen option). Notably, in our experimental setup unchosen reward probabilities were relevant for future predictions, but unchosen reward magnitudes were of no relevance because they changed randomly from trial to trial. Counterfactual prediction error coding in lFPC, DMFC, and PMC thus reflected the relevant information for learning about unchosen options in our task—reward probabilities. Consistent with the claim that lFPC, DMFC, and PMC encode counterfactual prediction errors, but not regret [25], activity in these regions was *not* sensitive to the difference between the reward magnitudes of obtained and unobtained outcomes. These data therefore constitute the first neural dissociation of counterfactual prediction errors from regret. Intriguingly, neurons that encode counterfactual rewards have recently been identified in the monkey dACC [12], which neighbors and is interconnected with the DMFC region identified here [39],[49] and is also interconnected with the PMC and FPC [38],[40]. These observations raise the possibility that unchosen reward signals in dACC might be integrated with unchosen expectations to compute counterfactual prediction errors in lFPC, DMFC, and PMC.

We also tested whether there exists a relationship between the neural coding of counterfactual prediction errors and the propensity to learn from counterfactual information. In the PMC there was a strong relationship between the effect of counterfactual prediction errors and how effectively participants learned from counterfactual outcomes. This finding suggests that neural coding of counterfactual information in PMC influences counterfactual learning behavior.

In neuroscience, there is an emerging view that predictive coding extends beyond the domain of experienced reward [18],[50],[51]. In the perceptual domain, unsigned prediction error (or surprise) responses have been identified in inferior temporal gyrus (ITG) when participants observe gabor patches whose orientation does not match the orientation of a template during A, not A decisions [18]. When the stimuli are faces or houses, rather than gabor patches, fusiform face area (FFA) and parahippocampal place area (PPA) are sensitive to unsigned prediction errors related to predictions concerning faces and houses, respectively [19],[22], a modulation that at least partly contributes to the phenomenon of repetition suppression in the FFA [19]. During incidental audio-visual learning, the BOLD response in primary visual cortex and putamen was shown to correlate with unsigned prediction errors, when the appearance (or absence) of a black and white shape stimulus was unpredicted (or predicted) by an auditory tone [20].

In the social domain, two recent investigations [15],[21] have revealed signed prediction error responses in the superior temporal sulcus (STS) and dorsomedial prefrontal cortex (DMPFC)—brain regions implicated in theory of mind tasks [52],[53]—when participants have to learn about the behavior of another individual. Prediction errors in these regions have been discovered when the objective was to learn about the reputation of a social partner [15], or when it was to learn about the influence of an opponent's choice on the likely future behavior of the opponent [21]. Collectively, these investigations in the perceptual and social domains carry fundamental implications: First, they suggest that prediction error coding is more ubiquitous than previously thought and, second, that brain regions specialized for a given class of information may also encode prediction errors specifically related to that class of information. The present finding that regions which encode information related to unchosen options also encode unchosen prediction errors adds counterfactual information to the classes of information for which prediction error signals have been identified.

In summary, we have delineated the functional contribution of a network centered on lFPC, DMFC, and PMC when human subjects decide between multiple alternatives. The results indicate that this network both forecasts reward expectations related to selecting untaken alternatives in the future and also updates those expectations—key computations for deciding and learning when to take the road less traveled.

## Materials and Methods

### Participants

Twenty-two healthy volunteers participated in the fMRI experiment. Two volunteers failed to use either the reward probabilities or reward magnitudes in the task, as indicated by values of nearly 0 for each of the free parameters in the behavioral model, and one volunteer failed to use reward probability, as indicated by values of 0 for both β and γ in the behavioral model (see Behavioral Model description below). These participants' data were therefore discarded from all analyses. The remaining 19 participants (10 women) were included in all further analyses. All participants gave informed consent in accordance with the National Health Service Oxfordshire Central Office for Research Ethics Committees (07/Q1603/11).

### Experimental Task

In our fMRI paradigm, participants decided repeatedly between three stimuli based on their expectation of reward and the number of points associated with each stimulus option (Figure 2A). Although the number of points was generated randomly and displayed on the screen, the expectation of reward had to be estimated from the recent outcome history. The three stimuli were pictures of a real face, whole body, and house. The identities of the face, body, and house were fixed for the duration of the experiment and across participants. During the first decision-making phase, the three options and their associated points were displayed at three locations on the screen: left, upper middle, and right. The location at which each stimulus was displayed was randomized across trials. When the yellow question mark appeared in the centre of the screen, participants indicated their choices with right-hand finger responses on a button box corresponding to the location of each stimulus. Immediately after participants indicated their choice, the first feedback phase was presented: the selected option was highlighted by a red rectangle that framed the chosen stimulus and the chosen outcome (reward or no reward) was presented. If the participant's choice was rewarded, a green tick appeared in the centre of the screen, and the red prize bar also updated toward the gold rectangular target in proportion to the amount of points won on that trial. Each time the prize bar reached the gold target, participants were rewarded with £2. If the participant's choice was not rewarded, a red X appeared in the centre of the screen, and the red prize bar remained stationary. These initial decision-making and chosen feedback phases were presented on every trial in the experiment.

After presentation of the chosen feedback, one of three different conditions followed in pseudorandom order. In condition 1 the *outcomes* for the two remaining unchosen options were presented. A green tick or a red X appeared on the left of the two options that were unchosen during the first decision-making phase, depending on whether they were rewarded or unrewarded. The red prize bar did not move. This event was followed by presentation of the next trial. This condition was critical because it enabled us to isolate activity during the first decision-making and feedback phases uncontaminated by activity related to a second decision. In conditions 2 and 3, participants had the opportunity to choose between the two remaining options that were unselected by the participant at the first decision. These two remaining stimuli maintained their spatial locations on the screen. In condition 2, the option reward probabilities and points associated with the two options remained identical to what they were at the first decision (Figure 2A). The purpose of this condition was to use the participants' responses at the time of the second decision to improve our ability to rank the two unchosen options at the time of the first decision on the basis of *expected value*. However, in condition 3 only the reward probabilities remained the same; the points for both remaining options were changed to 50 (Figure 2A). Therefore, the only information guiding participant decisions in condition 3 should theoretically be the reward probabilities. This condition was introduced to more accurately rank the two unchosen options at the first decision on the basis of *reward probability*. For both conditions 2 and 3, participants indicated their choice after a yellow question mark appeared. This was followed by simultaneous feedback for the chosen and unchosen options from the second decision. During this second feedback phase, a red rectangle framed the selected option and a green tick or red X was presented to the left of the chosen and unchosen options, depending on whether these options were rewarded or unrewarded. If the choice at the second decision was rewarded, the red prize bar updated in proportion to the number of points won. This event was followed by presentation of the next trial. There was no inter-trial interval in any condition. Each event was jittered between 2.5 and 5.5 s (uniform distribution). There were 60 trials in each condition, making 180 trials in total. Conditions were pseudorandomly interleaved and were uncued. Participants earned between £20 and £28 on the task, depending on their performance.

The true reward probabilities associated with each stimulus type varied independently from one trial to the next over the course of the experiment at a rate determined by the volatility, which was fixed in the current experiment. More specifically, the true reward probability of each stimulus was drawn independently from a beta distribution with a fixed variance and a mean that was determined by the true reward probability of that stimulus on the preceding trial. The true reward probabilities that participants tracked are shown in Figure S3.

### FMRI Data Acquisition and Analysis

FMRI data were acquired on a 3T Siemens TRIO scanner with a voxel resolution of 3×3×3 mm^3^, TR = 3 s, TE = 30 ms, Flip angle = 87°. The slice angle was set to 15° and a local z-shim was applied around the orbitofrontal cortex to minimize signal dropout in this region [54], which has previously been implicated in other aspects of decision making. The mean number of volumes acquired was 999, giving a mean total experiment time of approximately 50 min (see Text S1, Experimental Procedures for further details).

A general linear model (GLM) was fit in pre-whitened data space [55]. Twenty-four regressors were included in the GLM (see Table S1 for a summary): the main effect of the first decision-making phase; the main effect of the first feedback phase; the main effect of the foregone outcome phase (condition 1); the main effect of the second decision-making phase (conditions 2 and 3); the main effect of the second feedback phase (conditions 2 and 3); the interaction between chosen probability and the first decision-making phase; the interaction between chosen probability and the first feedback phase; the interaction between the best unchosen probability as determined by the model in conditions 1 and 2 and the first decision-making phase; the interaction between the best unchosen probability as determined by participant choices in condition 3 and the first decision-making phase; the interaction between the best unchosen probability as determined by the model in conditions 1 and 2 and the first feedback phase; the interaction between the best unchosen probability as determined by participant choices in condition 3 and the first feedback phase; the interaction between the worst unchosen probability as determined by the model in conditions 1 and 2 and the first decision-making phase; the interaction between the worst unchosen probability as determined by participant choices in condition 3 and the first decision-making phase; the interaction between the worst unchosen probability as determined by the model in conditions 1 and 2 and the first feedback phase; the interaction between the worst unchosen probability as determined by participant choices in condition 3 and the first feedback phase; the outcome at the first feedback phase; the outcome at the second feedback phase; and six motion regressors produced during realignment. Because there were not any notable differences between z-statistic maps based on the model or participant choices, we defined contrasts of parameter estimates (COPEs) for the best and worst unchosen probability as the combination of the regressors based on the model and participant choices. Based on the evidence from our previous investigation [8] that the lFPC encodes reward probability during both the decision and feedback phases, the reward probability regressors were modeled across both phases. To do so, additional COPEs defined the chosen, best unchosen, and worst unchosen probabilities as the sum of regressors over the first decision-making and feedback phases (Table S1).

For group analyses, EPI images were first registered to the high resolution structural image using 7 degrees of freedom and then to the standard [Montreal Neurological Institute (MNI)] space MNI152 template using affine registration with 12 degrees of freedom [56]. We then fit a GLM to estimate the group mean effects for the regressors described above. FMRIB's Local Analysis of Mixed Effects (FLAME) was used to perform a mixed effects group analysis [57],[58]. All reported fMRI z-statistics and *p*-values arose from these mixed effects analyses on all 19 participants. We report clusters of greater than 10 voxels that survived a threshold of z>3.1, *p*<0.001, uncorrected. It should be noted that our analyses carefully avoid selection bias in identifying regions related to probability. Based on the findings of our previous study and other investigations [8],[45],[46], we were confident that lFPC would encode the *relative* probability rather than either chosen or unchosen probability in isolation. One of the central aims of this experiment, however, was to test the hypothesis that the lFPC encoded the best unchosen probability *and* either the chosen probability, worst unchosen probability, or both (see Figure 1). For the probability-based analysis, rather than search for regions encoding the relative unchosen probability (e.g., the best unchosen probability relative to the chosen probability or the best unchosen probability relative to the average of the other probabilities), for which there are large effects in the lFPC (see Figure S1), we have searched only for regions that encode the best unchosen probability. We have used this analysis because it is orthogonal to the worst unchosen and chosen probability regressors and thus enables us to perform orthogonal tests on the regions of interest (ROIs) identified to test competing hypotheses. ROI analyses are presented in detail in Text S1, Experimental Procedures.

### Behavioral Model

We used an optimal Bayesian reinforcement-learning algorithm [13] to model participant estimates of the reward probabilities and their eventual choices. This model has been described in detail in previous investigations [8],[13],[15]. Briefly, the model is composed of a “predictor” that estimates the reward probability associated with each option and other environmental statistics given only the observed data (i.e., the reward outcomes of chosen and unchosen options) and a “selector” that chooses actions on the basis of these estimates. Because feedback is given on each option on each trial in our experimental task, the model updates the reward probability associated with each option upon receipt of feedback, as is optimal. These estimates of the reward probabilities were then combined with reward magnitude according to participant-specific free parameters that can differentially weigh probability, magnitude, and their product, to derive estimates of the subjective expected values.

We found no evidence that participants' choices at the first decision were influenced by the prospect of a second decision at which reward magnitudes could either remain the same or both change to 50 (Text S1). We therefore assumed that subjective value at both decisions was computed on the basis of the current decision alone:
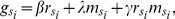
(1)where 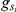, 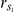, and 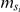 are the subjective value, reward probability, and reward magnitude associated with the stimulus (face, house, or body) on trial *i*. We fitted β, λ, and γ to each individual participant's behavioral data using standard non-linear minimization procedures implemented in Matlab 7 (Mathworks). Finally, the selector assumed that participants chose stimulus *s* according to the following softmax probability distribution:
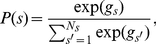
(2)where 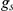 is the subjective expected value of the stimulus, and *N*_s_ is the total number of stimuli to choose between (*N*_s_ = 3 at the first decision, *N*_s_ = 2 at the second decision).

## Supporting Information

Figure S1 Relative unchosen probability. (A) Axial and coronal slices through z-statistic maps relating to the effect of the best unchosen reward probability minus the chosen reward probability. Activations are displayed at z>3.1, *p*<0.001, uncorrected, though left lFPC survives whole brain cluster correction at z>2.3, *p*<0.05.
(TIF)

Figure S2 Chosen reward prediction errors. (A) Axial slice through z-statistic map relating to the conjunction of the effects of chosen prediction error at decisions 1 and 2. Activations are displayed at z>3.1, *p*<0.01, cluster-corrected at the whole brain level. (B) Time course from an ROI centered on the maximum of left ventral striatum showing a positive correlation with outcome (reward or no reward) and a negative correlation with chosen reward probability in response to presentation of feedback on the chosen option.
(TIF)

Figure S3 True reward probabilities. The true reward probability that generated actual rewards is shown for faces, bodies, and houses in cyan, pink, and purple, respectively.
(TIF)

Figure S4 Comparison of actual choice frequencies and model-based choice probabilities. Top row: Group mean ± SEM for choices of the best option is plotted against the optimal choice probability as predicted by the Bayesian model for decisions 1 (left) and 2 (right). Bottom row: Group mean ± SEM for choices of the second best option is plotted against the model-based probability of choosing the second best option for decisions 1 (left) and 2 (right). Participants chose between three options at decision 1 and two options at decision 2.
(TIF)

Table S1 Summary of interactions and contrasts included in the design matrix.
(DOC)

Table S2 Activated clusters resulting from the whole-brain analysis, for the interactions of interest.
(DOC)

Text S1 Supplemental data and supplemental experimental procedures provide further details of the experimental task and analyses.
(DOC)

## Abbreviations

COPE: contrasts of parameter estimate
dACC: dorsal anterior cingulate cortex
DMFC: dorsomedial frontal cortex
FFA: fusiform face area
FLAME: FMRIB's Local Analysis of Mixed Effects
lFPC: lateral frontal polar cortex
ITG: inferior temporal gyrus
GLM: general linear model
PMC: posteromedial cortex
PPA: parahippocampal place area
RL: reinforcement learning
ROIs: regions of interest
STS: superior temporal sulcus
VMPFC: ventromedial prefrontal cortex
