# Supplementary figures and images for: Counterfactual Choice and Learning in a Neural Network Centered on Human Lateral Frontopolar Cortex
Source: PLoS Biol. 2011 Jun 28;9(6):e1001093. doi: 10.1371/journal.pbio.1001093 (PMC3125157; doi:10.1371/journal.pbio.1001093)

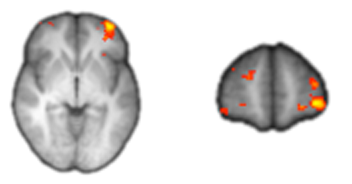

Supplement: Figure S1 — Relative unchosen probability. (A) Axial and coronal slices through z-statistic maps relating to the effect of the best unchosen reward probability minus the chosen reward probability. Activations are displayed at z>3.1, p<0.001, uncorrected, though left lFPC survives whole brain cluster correction at z>2.3, p<0.05. (TIF) [file pbio.1001093.s001.tif]

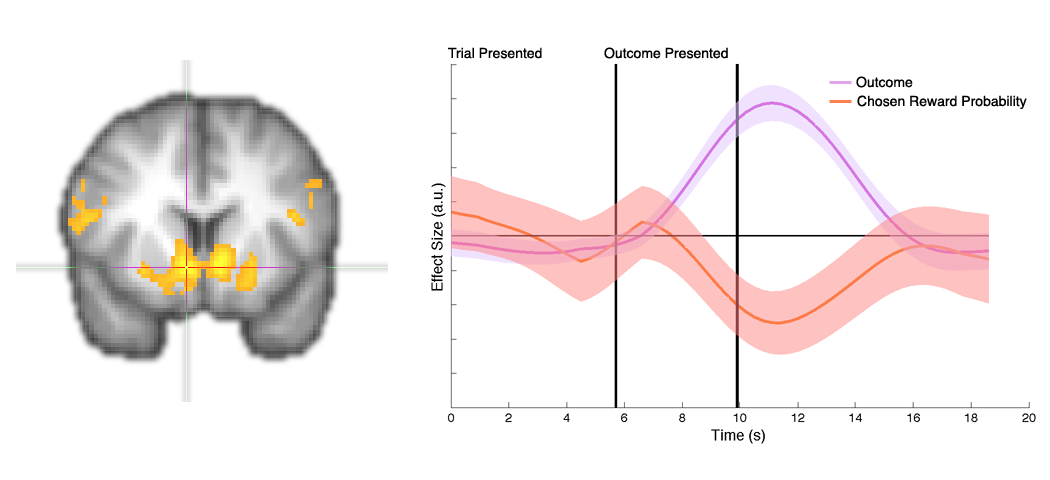

Supplement: Figure S2 — Chosen reward prediction errors. (A) Axial slice through z-statistic map relating to the conjunction of the effects of chosen prediction error at decisions 1 and 2. Activations are displayed at z>3.1, p<0.01, cluster-corrected at the whole brain level. (B) Time course from an ROI centered on the maximum of left ventral striatum showing a positive correlation with outcome (reward or no reward) and a negative correlation with chosen reward probability in response to presentation of feedback on the chosen option. (TIF) [file pbio.1001093.s002.tif]

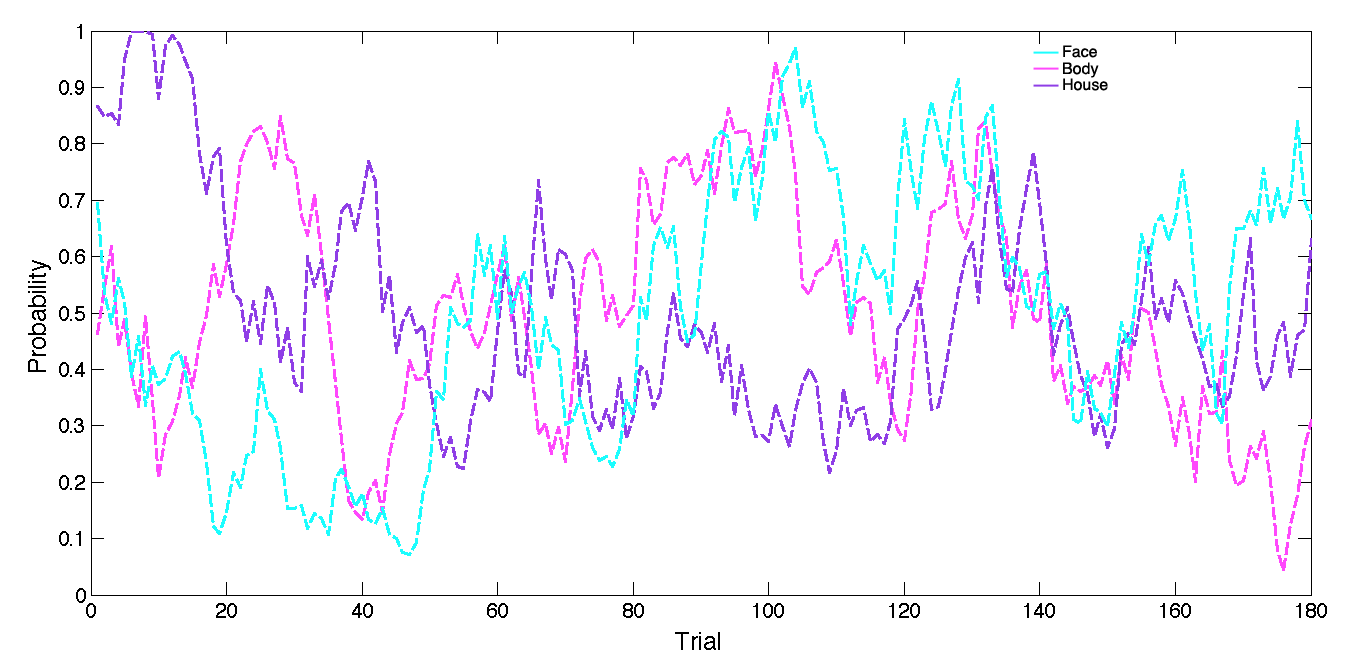

Supplement: Figure S3 — True reward probabilities. The true reward probability that generated actual rewards is shown for faces, bodies, and houses in cyan, pink, and purple, respectively. (TIF) [file pbio.1001093.s003.tif]

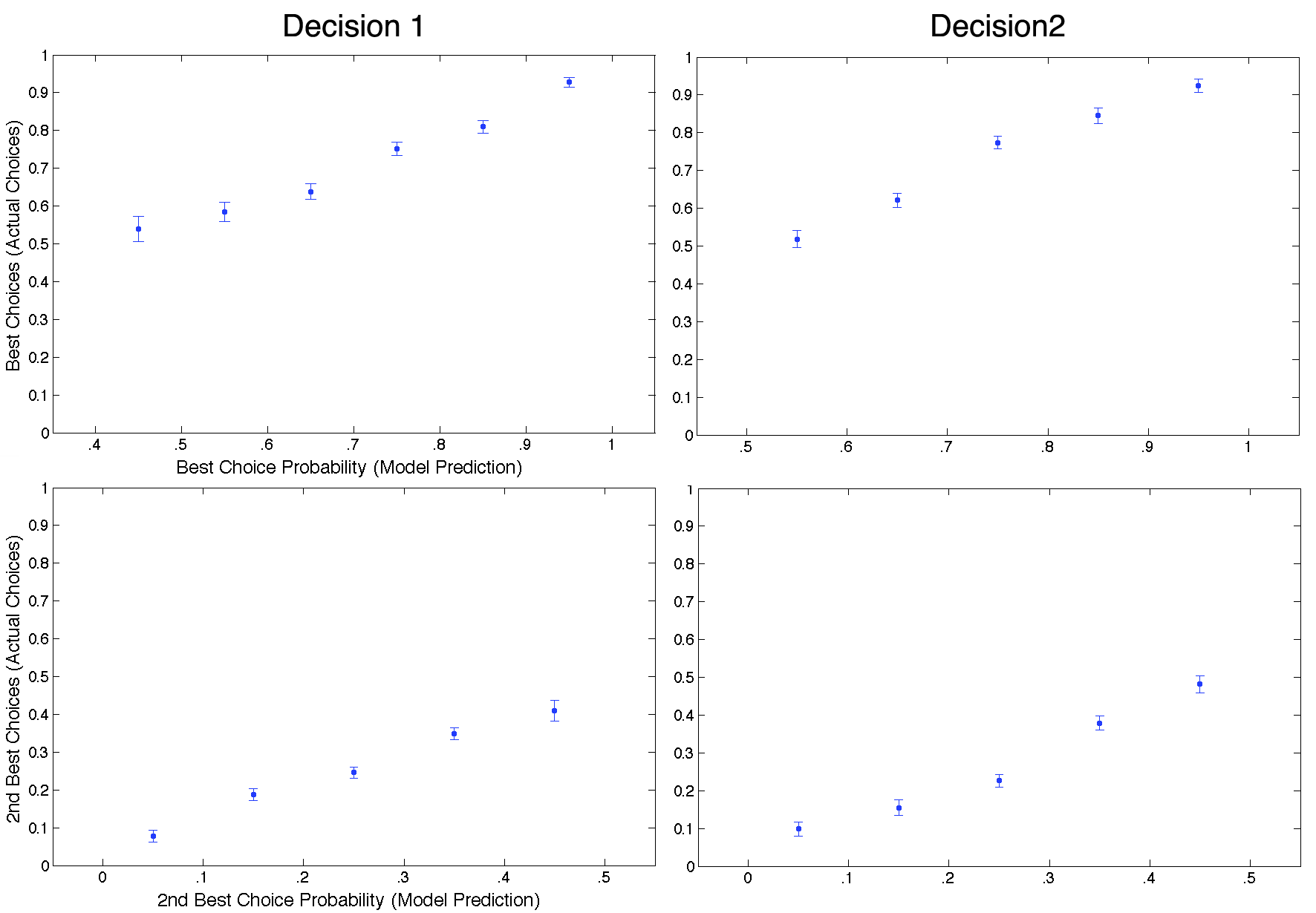

Supplement: Figure S4 — Comparison of actual choice frequencies and model-based choice probabilities. Top row: Group mean ± SEM for choices of the best option is plotted against the optimal choice probability as predicted by the Bayesian model for decisions 1 (left) and 2 (right). Bottom row: Group mean ± SEM for choices of the second best option is plotted against the model-based probability of choosing the second best option for decisions 1 (left) and 2 (right). Participants chose between three options at decision 1 and two options at decision 2. (TIF) [file pbio.1001093.s004.tif]
