# Supplementary material for: Counterfactual Choice and Learning in a Neural Network Centered on Human Lateral Frontopolar Cortex
Source: PLoS Biol. 2011 Jun 28;9(6):e1001093. doi: 10.1371/journal.pbio.1001093 (PMC3125157; doi:10.1371/journal.pbio.1001093)
Supplement: Table S1 — Summary of interactions and contrasts included in the design matrix. (DOC) [file pbio.1001093.s005.doc]

| Explanatory Variables | Decide 1 Phase | Feedback 1  Phase | Foregone Phase | Decide 2  Phase | Feedback 2  Phase | Contrast1 | Contrast2 |
| --- | --- | --- | --- | --- | --- | --- | --- |
| Chosen Probability | X | X |  |  |  |  |  |
| Best Unchosen  Probability (based on subject choices) | X | X |  |  |  | X |  |
| Best Unchosen  Probability (based on the model) | X | X |  |  |  | X |  |
| Worst Unchosen Probability (based on subject choices) |  |  |  |  |  |  | X |
| Worst Unchosen Probability (based on the model) |  |  |  |  |  |  | X |
| Prediction error of decision 1 |  | X |  |  |  |  |  |
| Prediction error of decision 2 |  |  |  |  | X |  |  |
