# Supplementary material for: Counterfactual Choice and Learning in a Neural Network Centered on Human Lateral Frontopolar Cortex
Source: PLoS Biol. 2011 Jun 28;9(6):e1001093. doi: 10.1371/journal.pbio.1001093 (PMC3125157; doi:10.1371/journal.pbio.1001093)
Supplement: Table S2 — Activated clusters resulting from the whole-brain analysis, for the interactions of interest. (DOC) [file pbio.1001093.s006.doc]

| Comparison | Anatomical Region | Cluster Extent (voxels) | Hemisphere | Peak Coordinates (mm) (x, y, z) | Maximum z score |
| --- | --- | --- | --- | --- | --- |
| Best Unchosen Probability (pending option with the highest reward probability) during the decision and feedback phases | lFPC | 22  11 | L  L | -36, 58, -4  -32, 46, -2 | 3.50  3.64 |
|  | DMFC | 14 | R | 6, 34, 42 | 3.33 |
|  | PMC | 93 | R | 2, -62, 38 | 3.70 |
| Experiential Reward Prediction Error | Lateral Occipital Cortex (Inferior) | 3853 | L | -44, -74, -6 | 4.82 |
|  | Lateral Occipital Cortex (Inferior) | 2884 | R | 48, -62, -12 | 5.06 |
|  | Ventral Striatum (Nucleus Accumbens) | 1803 | R | 8, 12, -6 | 5.03 |
|  | Supramarginal Gyrus (Anterior) | 1186 | R | 62, -26, 38 | 4.46 |
|  | Ventrolateral Prefrontal Cortex (Posterior) | 334 | L | -38, 4, 22 | 4.07 |
|  | Ventrolateral Prefrontal Cortex (Posterior) | 304 | R | 52, 6, 20 | 4.4 |
|  | Posterior Cingulate Cortex | 287 | N/A | 0, -38, 34 | 3.97 |
|  | Ventrolateral Prefrontal Cortex (Anterior) | 167 | L | -46, 46, 2 | 3.68 |
