# Supplementary material for: Counterfactual Choice and Learning in a Neural Network Centered on Human Lateral Frontopolar Cortex
Source: PLoS Biol. 2011 Jun 28;9(6):e1001093. doi: 10.1371/journal.pbio.1001093 (PMC3125157; doi:10.1371/journal.pbio.1001093)
Supplement: Text S1 — Supplemental data and supplemental experimental procedures provide further details of the experimental task and analyses. (DOC) [file pbio.1001093.s007.doc]

**Supplementary Information**

**Supplemental Data**

To confirm that the significant negative effect of the worst option on choosing the best option was not driven by misclassification of mid and worst options by the model on some trials, these analyses were repeated for choices made in condition 2 alone. Importantly, introducing condition 2 enabled us to classify which option the subject ranked as the mid and worst options in terms of *value* at the first decision on the basis of their choices at the second decision (see Figure 2A). Restricting the logistic regression to these trials upheld the results from the analysis based on trials from all conditions: there was a robust positive effect of the best option (reward probability: t(4)= 5.33, p<0.0001; reward magnitude: t(4)= 3.18, p= 0.0026), a robust negative effect of the mid option (reward probability: t(4)=-4.30, p= 0.0002; reward magnitude: t(4)=-3.85, p=0.0006), and a modest but significant negative effect of the worst option (reward probability: t(4)=-2.23, p=0.02; reward magnitude: t(4)=-2.29, p=0.017) on choosing the best option.

**Supplemental Experimental Procedures**

*FMRI*

We acquired Field Maps using a dual echo 2D gradient echo sequence with echos at 5.19 and 7.65 ms, and repetition time of 444ms. Data were acquired on a 64x64x40 grid, with a voxel resolution of 3mm isotropic. T1-weighted structural images were acquired for subject alignment using an MPRAGE sequence with the following parameters: Voxel resolution 1x1x1 mm^3^ on a 176x192x192 grid, Echo time(TE)= 4.53 ms, Inversion time(TI)= 900 ms, Repetition time (TR)= 2200 ms.

FMRI analysis was carried out using FMRIB’s Software Library (FSL) . Data were preprocessed using the default options in FSL: motion correction was applied using rigid body registration to the central volume ; Gaussian spatial smoothing was applied with a full width half maximum of 5mm; brain matter was segmented from non-brain using a mesh deformation approach ; high pass temporal filtering was applied using a Gaussian-weighted running lines filter, with a 3dB cutoff of 100s.

Aside from the motion regressors, all regressors were convolved with the FSL default hemodynamic response function (Gamma function, delay=6s, standard deviation=3s), and filtered by the same high pass filter as the data. Temporal derivatives were excluded from the GLMs because they induced correlations with some of the task regressors.

ROI analyses were conducted on clusters of activation identified from the whole-brain voxelwise analysis in order to perform orthogonal statistical tests and characterize the time course of BOLD fluctuations in these regions. In each subject we extracted and averaged pre-whitened and filtered BOLD data from a 3cm radius sphere centered on the group maximum back-projected from the ROIs identified by the group analyses.

Each subject’s BOLD timeseries was then divided into trials, which were resampled to 300ms and truncated based on the mean trial length for each condition across trials and subjects. In the resampled timeseries, trial events were aligned based on their mean onset times across trials and individuals. A general linear model was then fit across trials in each subject independently. We then calculated group mean effect sizes at each time point, and their standard errors. Data and regressors were Z-normalized so that effect sizes could be reported as (partial) correlations. To ascertain which variables were reflected in BOLD activity in a given ROI, we then fit the BOLD effect of interest with a hemodynamic response function (double gamma function) aligned to the onset of the event in each subject.

To produce scatterplots in Fig. 5B, we identified the effect size in each subject at the time of the first group peak in the effect size from condition 1 (i.e., on the subset of trials on which there was not a second decision that could interfere with the signal). The corresponding effect size for the relative unchosen probability in the FPC was then plotted against the proportion of trials on which subjects chose P2 at the second decisions. For the scatterplot in Figure 6C, we similarly identified the effect size for the counterfactual prediction error in each subject at the time of the group peak. This was then plotted against the difference in fits between the optimal model and the experiential model in each subject (see Behavioral Model description).

*Behavior*

*Alternative models*

We initially considered the possibility that participants took into account the likelihood of encountering a second decision when making initial decisions. In our experimental setup one of three events could pseudorandomly follow feedback for the initial decision: foregone feedback for the unselected options, a second decision in which reward magnitudes remained the same as at the first decision, or a second decision in which reward magnitudes both changed to 50. Hence, it would be advantageous to consider that on two-thirds of trials (although subjects did not know this proportion), a second decision would be encountered in which one of two things could happen: reward magnitudes of the two remaining options could remain the same or they could both change to 50. To attempt to account for this feature of the experimental design, we considered the possibility that subjects averaged across the two possible scenarios, weighted by their estimate of the likelihood that a second decision would be encountered:

$\begin{matrix} g_{sci}=\beta r_{sci}+\lambda m_{sci}+\gamma r_{sci}m_{sci}+ \\ \tau\left[\frac{(\beta r_{sbi}+\lambda m_{sbi}+\gamma r_{sbi}m_{sbi})+(\beta r_{swi}+\lambda m_{swi}+\gamma r_{swi}m_{swi})+(\beta r_{sbi}+\lambda50_{sbi}+\gamma r_{sbi}50_{sbi})+(\beta\cdot r_{swi}+\lambda50_{swi}+\gamma r_{swi}50_{swi})}{2}\right], \end{matrix}$where$g_{sc_{i}}$, $r_{sc_{i}}$, $m_{sc_{i}}$ are the subjective value, reward probability, and reward magnitude associated with the chosen stimulus at trial *i*, and $r_{sb_{i}}$and $m_{sb_{i}}$are the reward probability and reward magnitude associated with the best unchosen stimulus and $r_{sw_{i}}$and $m_{sw_{i}}$are the reward probability and reward magnitude associated with the worst unchosen stimulus at trial *i*. Therefore τ reflects the weight attributed to the possibility of encountering a second decision. We also considered the possibility that subjects instead computed the maximum of the two remaining options not selected at the first decision in each scenario (conditions 2 and 3) and averaged across the two scenarios:

$$g_{sci}=\beta r_{sci}+\lambda m_{sci}+\gamma r_{sci}m_{sci}+\tau\left[\frac{(\beta r_{sbi}+\lambda m_{sbi}+\gamma r_{sbi}m_{sbi})+(\beta r_{sbi}+\lambda50_{sbi}+\gamma r_{sbi}50_{sbi})}{2}\right].$$

We found no evidence that participants’ choices at the first decision were influenced by the prospect of a second decision at which reward magnitudes could either remain the same or both change to 50: values of τ were equal to 0 or nearly 0 in every participant.

We also compared the Bayesian model with a standard Rescorla Wagner RL model. The algorithm learns a reward probability *r*_t_(*s*) for each stimulus *s*, which is then updated on every trial, independently of whether or not stimulus *s* is chosen (as feedback is given for each option on every trial) by *r*_t+1_(*s*) = *r*_t_(*s*) + *αδ_t_*. The prediction error *δ_t_* = *p_t_* - *r*_t_(*s*) is the discrepancy between the predicted reward *r*_t_(*s*) and obtained reward *p_t_*.

Next we constructed a similar model but with two separate learning rates, one for chosen stimuli and one for counterfactual stimuli, to test whether there is evidence whether people learn more effectively from chosen and counterfactual feedback. Thus, if a stimulus was chosen, it was updated according to *r*_t+1_(*s*) = *r*_t_(*s*) + *αδ_t_*. If it was not chosen, it was updated according to *r*_t+1_(*s*) = *r*_t_(*s*) + *ηδ_t_.* Comparison of the subject-specific best-fitting values of the parameters of *α* and *η* for the pool of participants tested did not reveal any significant difference (t(18)<.25, p>0.4).

Reward probabilities learned according to both models described above were then combined with reward magnitudes to compute subjective expected values in the same way as described earlier:

$$g_{sci}=\beta r_{sci}+\lambda m_{sci}+\gamma r_{sci}m_{sci}$$

Once again, the selector assumed that subjects chose stimulus *s* according to the following softmax probability distribution:

$P(s)=\frac{\exp(g_{s})}{\sum_{s'=1}^{N_{s}} \exp(g_{s'})},$ (e*q 2*)

where $g_{s}$is the subjective expected value of the stimulus, and *N*_s_ is the total number of stimuli to choose between (*N*_s_=3 at the first decision, *N*_s_=2 at the second decision).

*Logistic regression analysis*

For our logistic regression model of choice behavior, optimal versus suboptimal choices were the dependent variable and the reward probability and reward magnitude of the best, mid and worst options constituted the six independent variables.

*Experimental Task*

To decorrelate the reward probabilities, reward magnitudes, and expected values associated with each option, and between simulated chosen and unchosen options, we used a simple genetic algorithm with 1000 generations that aimed to reduce the summed absolute value of correlations between our variables of interest (see Results; Figure 3A). We simulated choices using both greedy and softmax action selection rules , inserting into the softmax the inverse temperature fitted to subject choices from a previous study . This procedure gave us some insight into the extent to which chosen, best unchosen, and worst unchosen variables would also be de-correlated.

**Supplemental References**

1. Smith SM, Jenkinson M, Woolrich MW, Beckmann CF, Behrens TE, et al. (2004) Advances in functional and structural MR image analysis and implementation as FSL. Neuroimage 23 Suppl 1: S208-219.

2. Jenkinson M, Bannister P, Brady M, Smith S (2002) Improved optimization for the robust and accurate linear registration and motion correction of brain images. Neuroimage 17: 825-841.

3. Smith SM (2002) Fast robust automated brain extraction. Hum Brain Mapp 17: 143-155.

4. Sutton RS, Barto AG (1998) Reinforcement Learning: An Introduction: MIT Press, Cambridge, Massachusetts.

5. Boorman ED, Behrens TE, Woolrich MW, Rushworth MF (2009) How green is the grass on the other side? Frontopolar cortex and the evidence in favor of alternative courses of action. Neuron 62: 733-743.
